# The complete chloroplast genome of *Liparis brunnea* Ormerod (Orchidaceae)

**DOI:** 10.1080/23802359.2024.2432352

**Published:** 2025-12-05

**Authors:** Xin-jun Gan, Chu-qiao Tang, Pei-qi Yu, Huan-jin Chen, Jiu-xiang Huang

**Affiliations:** ^a^ Management Office of Chenhedong Provincial Nature Reserve, Conghua, China; ^b^ College of Forestry and Landscape Architecture, School of South China Agricultural University, Guangzhou, China

**Keywords:** Chloroplast genome, phylogenetic analysis, *Liparis brunnea*

## Abstract

*Liparis brunnea* Ormerod,a herbaceous species native to China, has a complete chloroplast genome of 150,145 bp (GC 36.90%), exhibiting a typical LSC (84,600 bp), SSC (15,701 bp) and IR (24,922 bp) structure. The plastome contains 107 genes (73 protein-coding genes, 30 tRNA genes and 4 rRNA genes). Phylogenetic analysis of 18 Malaxideae chloroplast genomes indicates that *L. brunnea* is closely related to *L. auriculata*. This study supports future population genetics and diversity studies.

## 1. Introduction

The genus *Liparis* Richard comprises approximately 400 perennial herbaceous species, with about 60 species in China and a widely distribution in tropical subtropical regions of Asia, Africa and South America (http://www.the plantlist.org) (Chen et al. 2009; Li et al. 2020). Recently, many phylogenetic studies of the genus *Liparis* were performed using different molecular fragments (Tang et al. 2015). The results indicated that *Liparis* is polyphyletic with its species being classified into two groups: the terrestrial group and the epiphytic group. However, its close relatives, such as *Oberonia*, *Crepidium* and *Dienia,* etc., are nested within *Liparis*. Subsequent investigations have proposed subdivision of epiphytic *Liparis* into the genera *Cestichis*, *Stichorkis*, *Platystyliparis* and *Alatiliparis* (Margońska and Szlachetko 2001). However, this taxonomic revision has not been widely accepted due to incomplete species sampling and limited gene fragment data (Li et al. 2020).

*Liparis brunnea* Ormerod (Ormerod 2007) is an epiphytic herb that grows on rockets covered in damp moss. It primarily occurs in Guangdong, Guangxi, Hunan and Sichuan province in China (Huang et al. 2023; Xiao et al. 2023; Zheng et al. 2023). The leaves of *L. brunnea* are ovate-elliptic to suborbicular, measuring 10–17.5 × 7–11 mm, with a subacute apex and a base that contracts into a sheath. The flowers are brown with reflexed linear dorsal sepals approximately 8.3 × 0.7–0.8 mm, and subquadrate, emarginate labella around 8.5 × 7 mm. featuring a deeply bilobed callus at the base. The column is slender, arcuate and narrowly winged apically (Ormerod 2007).

However, the phylogentic position of *L. brunnea* is still enigmatic. The chloroplast genome is commonly characterized by a highly conserved structure and a relatively low rate of nucleotide substitution (Wicke et al. 2011). Consequently, it has been extensively utilized for resolving the phylogenetic relationships among various plant lineages (Li et al. 2021). In this study, we employed high-throughput sequencing technology to assemble and annotate the chloroplast genome of *L. brunnea*, enabling us to clarify the phylogenetic position of *L. brunnea*. The molecular data presented in this study serve as a foundational resource for investigating the phylogeny and population genetics of *Liparis*.

## 2. Materials and methods

### 2.1. Plant materials

**Figure 1.**
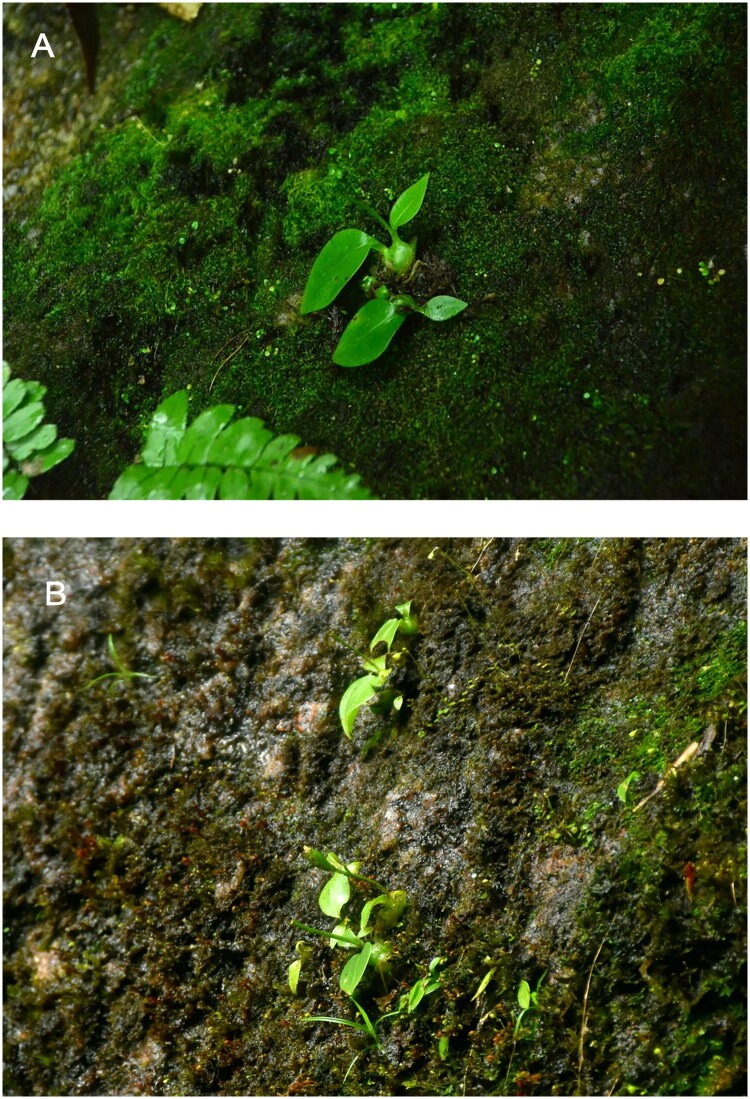
Species reference image of *L. brunnea.* This photo was taken by Yuling Li with the author’s approval for use. The leaves are ovate-elliptic to suborbicular with subacute, while the flowers are brown with linear dorsal sepals and subquadrate and emarginate labella.

The plant materials of *L. brunnea* sequenced in this study were collected from Conghua District, Guangzhou (Figure 1) at longitude 23°45′1.75″ and latitude 113°55′57.32″. The fresh and healthy leaves were dried using silica gel and preserved for DNA extraction. A specimen was deposited at the Herbarium of South China Agricultural University (CANT, Yongbin Wu, ybwu@scau.edu.cn) under the voucher number HJX2023052501.

### 2.2. Methods

Total DNA was extracted from fresh and healthy leaves using CTAB method (Doyle and Doyle 1987). DNA degradation and contamination were monitored on 1% agarose gels. DNA purity was determined with the NanoPhotometer^®^ spectrophotometer (Implen, CA, USA), and DNA concentration was measured using the Qubit^®^ DNA Assay Kit in a Qubit^®^ 2.0 Fluorometer (Life Technologies, CA, USA). The qualified DNA was fragmented by Covaris M220 Focused-ultrasonicator (Covaris, MA) instrument. The fragmented DNA was repaired at the end, followed by the addition of the sequencing adapter, and then the ∼400 bp fragments of the genome were enriched through magnetic beads adsorption and amplified by PCR to form sequencing library. The libraries that passed the quality inspection were sequenced using the Illumina HiSeq 4000 platform according to the manufacturer’s instructions, and 150 bp paired-end reads were generated. To obtain clean data, the adaptor and low quality sequences were removed by Fastq 0.19.6 software (Chen et al. 2018).

The GetOrganelle pipeline software (Jin et al. 2020) was utilized for the assembly of the whole chloroplast genome. Subsequently, the assembled chloroplast genomes were visually inspected and edited using Bangdage (Wick et al. 2015). A complete circular genome was then generated for this sample. The annotation of chloroplast genome of *L. brunnea* was performed using PGA (Plastid Genome Annotator) (Qu et al. 2019), with reference to the plastome of *L. auriculata* (Kim YK et al. 2020). Visual inspection and manual editing were performed as necessary in Geneious v 11.1.5 (Kearse et al. 2012). Ultimately, a high-quality and complete chloroplast genome sequence of *L. brunnea* was obtained. CPGAVAS2 (Shi et al. 2019) was used to visualize the structure features of the plastomes of *L. brunnea.*

The phylogenetic tree was constructed using the complete chloroplast genome sequences of *L. brunnea* and 12 other *Liparis* species, with five species from allied genera *Oberonia*, *Bulbophyllum* and *Dendrobium* was used as the outgroup. All the 18 chloroplast genomes were aligned by Muscle v3.8.31 (Edgar 2004) with the default settings and manually refined. The phylogenetic tree was generated by IQ-TREE v2.0.3 (Nguyen et al. 2015) with 1000 bootstrap (BS) replicates.

## 3. Results

**Figure 2.**
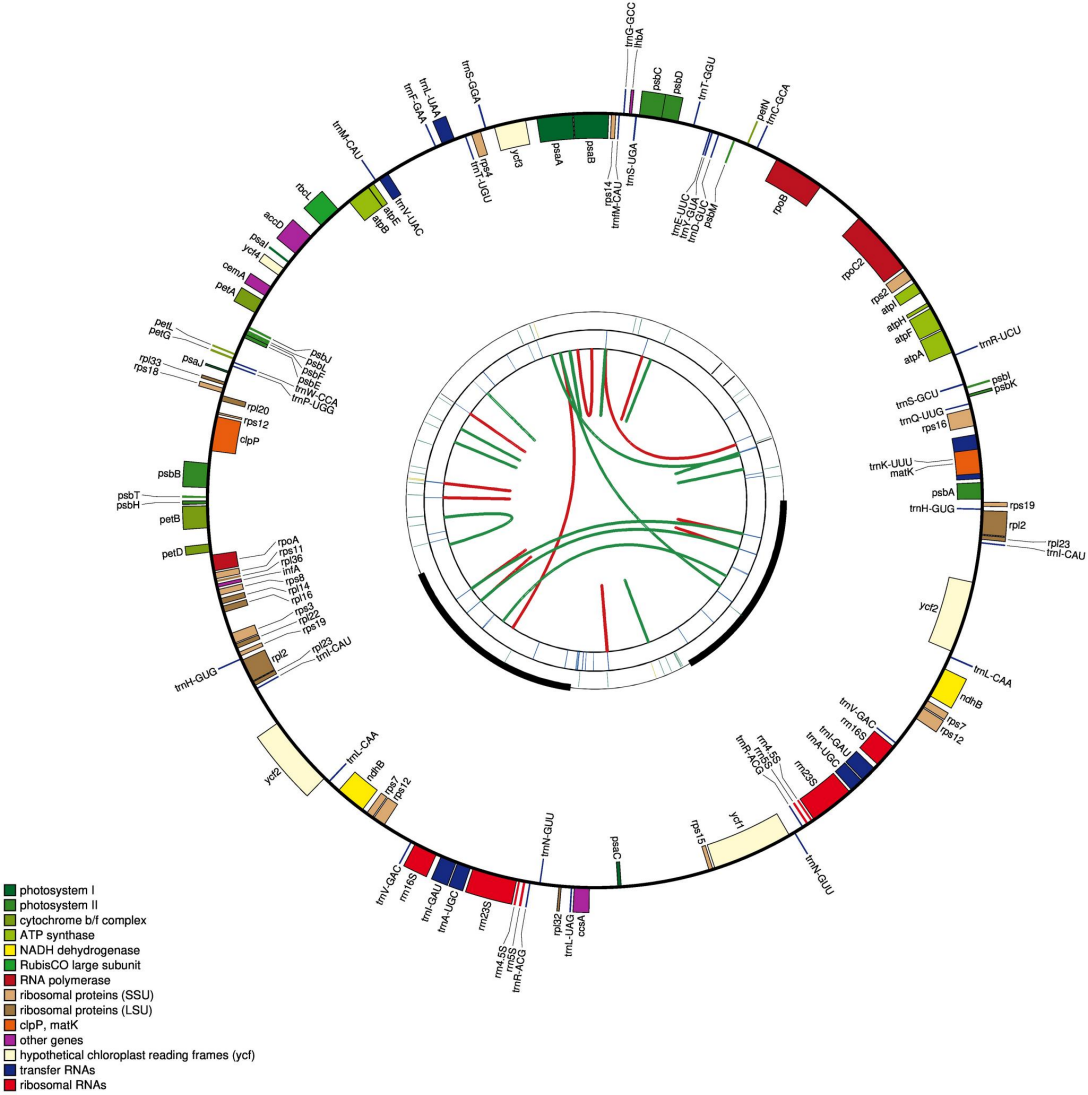
Circular map of the complete chloroplast genome of *L. brunnea* by CPGAVAS2. From the inside out, the first circle shows the forward and reverse repeats connected with red and green arcs. The second and third circles show the tandem repeats and microsatellite sequences marked with short bars. The outer circle shows the gene structure of the chloroplast genome. Genes have been colored according to their functional categories, shown in the lower left corner.

The complete chloroplast genome of *L. brunnea* (Figure 2) has a total length of 150,145 bp, which is consistent with the typical structure found in most chloroplast genomes. It exhibits a minimum read mapping depth of 207 x and an average read mapping depth of 870 x (Supplementary Figure 1). The genome consists of three distinct regions: a large single copy region (LSC) spanning 84,600 bp, a small single copy region (SSC) spanning 15,701 bp, and two inverted repeat regions (IR) spanning 24,922 bp each. The overall GC content is measured at 36.9%, while the LSC, SSC, and IR regions have GC contents of approximately 34.5%, 29.5%, and 43.5% respectively. Our annotation indicated that total of 126 genes (107 unique) were identified within the chloroplast genome, including 80 protein-coding genes (CDS) (73 unique), 38 tRNA (30 unique) genes and 8 rRNA genes (4 unique). Among these CDS, there are nine genes involved in Cis-splicing genes (Supplementary Figure 2(a)), while the structure of the trans-splicing gene was depicted (Supplementary Figure 2(b)). Additionally, *ndhA, ndhD, ndhF, ndhH, ndhJ, psbN* and *ycf15* genes were missing.

**Figure 3.**
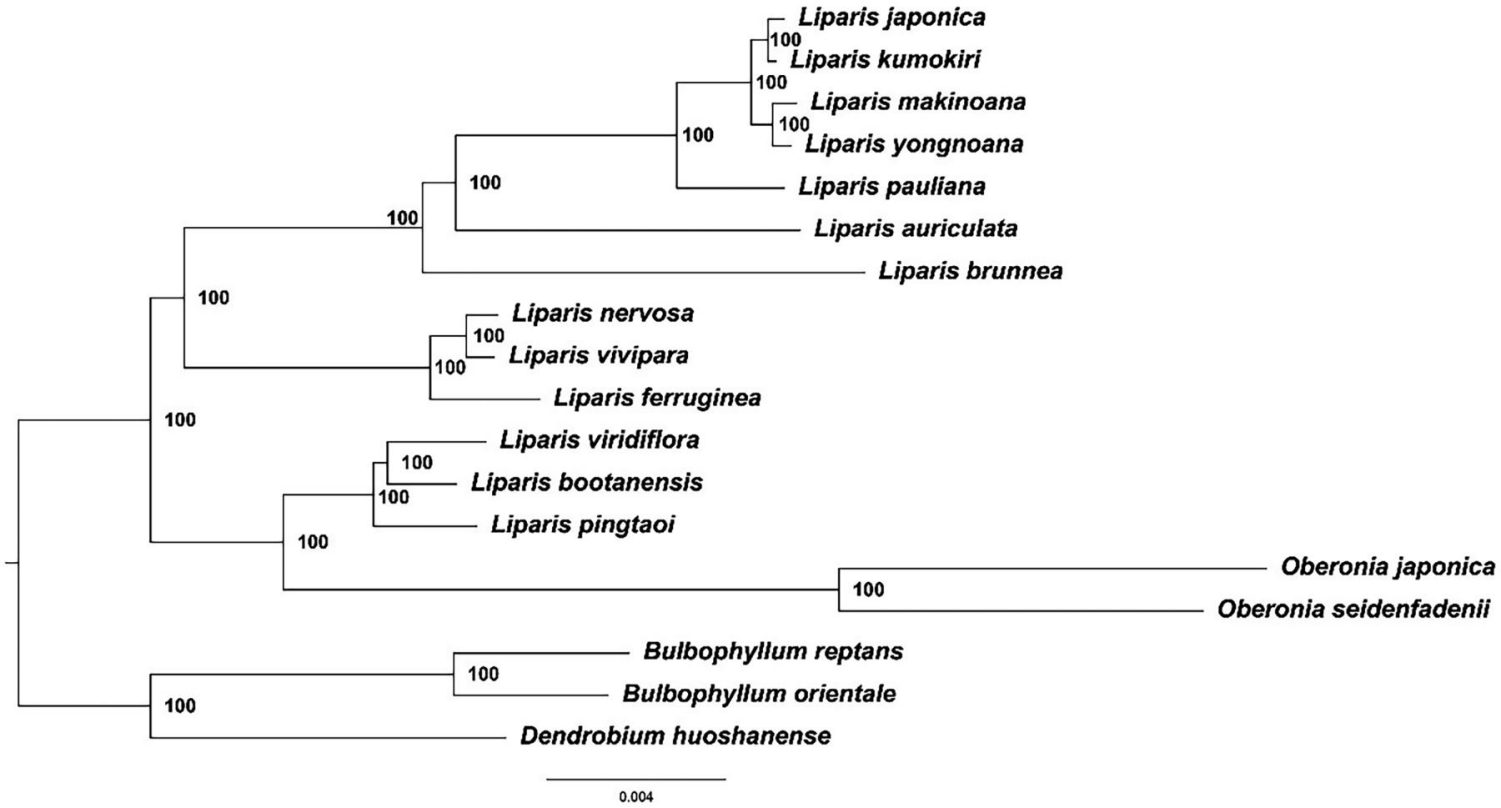
The ML phylogenetic tree based on the complete genome sequences of *L. brunnea* and 17 other species. The following sequences were used: *Liparis japonica* (MK886513) (Li et al. 2019), *Liparis kumokiri* (MN687902) (Chen et al. 2023), *Liparis makinoana* (MN686020) (Chen et al. 2023), *Liparis yongnoana* (MK801140) (Ha et al. 2019), *Liparis pauliana* (MN686022) (Chen et al. 2023), *Liparis auriculata* (NC046800) (Kim et al. 2020), *Liparis nervosa* (MN480463) (Wang et al. 2019), *Liparis vivipara* (MK862100) (Zhang et al. 2019), *Liparis viridiflora* (NC063587) (Chen 2023) *Liparis bootanensis* (NC047433) (Liu 2020), In addition, the following sequences were used as outgroup: *Bulbophyllum reptans* (LC642726) (Yang et al. 2022), *Bulbophyllum Orientale* (LC642725) (Yang et al. 2022), *Oberonia japonica* (KX871235) (Kim et al. 2020), *Oberonia seidenfadenll* (MN414241) (Jiang et al. 2019).

Based on the whole chloroplast genome dataset, we constructed a highly resolved phylogenetic tree comprising 18 species (Figure 3), with all the branches exhibiting robust support values (BS = 100). The phylogenetic analysis revealed that *L. brunnea* was locate at the base of Clade 1 with a strong support (BS = 100), which was closely related to *L. auriculata*. Within Clade 3, *L. viridiflora*, *L. bootanensis* and *L. pingtaoi* were sister to *Oberonia* species also with a strong support (BS = 100).

## 4. Discussion and conclusion

The size of published Orchidaceae chloroplast genomes ranged from 30,464 bp (*Gastrodia longistyla*) (Yang 2007; Guo et al. 2021) to 207,142 bp (Comparative Chloroplast Genomics of Seven Endangered Cypripedium Species and Phylogenetic Relationships of Orchidaceae). In this study, the length of the assembled *L. brunnea* chloroplast genome was determined to be 150,145 bp with the GC content of 36.9%. The structure of the chloroplast genome in *L. brunnea* is consistent with other terrestrial *Liparis* species and exhibits a typical quadripartite structure. However, its sequence length is approximately 3,000 bp shorter due to the absence of the *ndhF* sequence when compared through sequence comparison. The mechanism behind this gene deletion remains unclear.

To determine the phylogenetic position of *L. brunnea* within *Liparis*, our research focused on constructing phylogenetic trees based on 18 whole chloroplast genomes using the maximum likelihood method (ML). The results were similar to those obtained by Li et al. (2020) and Tang et al. (2015), where *Liparis* was divided into two group. Meanwhile, the *Liparis* species which locate in the clade 3 group formed a branch with *Oberonia*, indicating that *Liparis* is not a monophyletic group. *L. brunnea* was found at the base of clade 1 and forms a sister group with other clade 1 species branch with strong support rate (BS = 100) in our analysis. Due to limited taxon sampling, it is challenging to accurately reflect phylogenetic relationships among all the *Liparis* species. In this study, we have successfully sequenced and assembled the chloroplast genome of *L. brunnea* which complements existing molecular data gaps for this species and provides essential information for future studies on *Liparis* phylogenomic analysis as well as population genetics research on *L. brunnea*.

## Supplementary Material

Supplementary Figure 1 Read coverage plot.jpg

## Data Availability

The genome sequence data that support the findings of this study are openly available in GenBank of NCBI at (https://www.ncbi.nlm.nih.gov) under the accession no. PP140668. The associated **BioProject**, **SRA**, and **Bio-Sample** numbers are PRJNA1097209, SRR28578119, and SAMN40861830 respectively.
