# Supplementary figures and images for: The complete chloroplast genome of Liparis brunnea Ormerod (Orchidaceae)
Source: Mitochondrial DNA B Resour. 2025 Dec 5;11(1):28–32. doi: 10.1080/23802359.2024.2432352 (PMC12683749; doi:10.1080/23802359.2024.2432352)

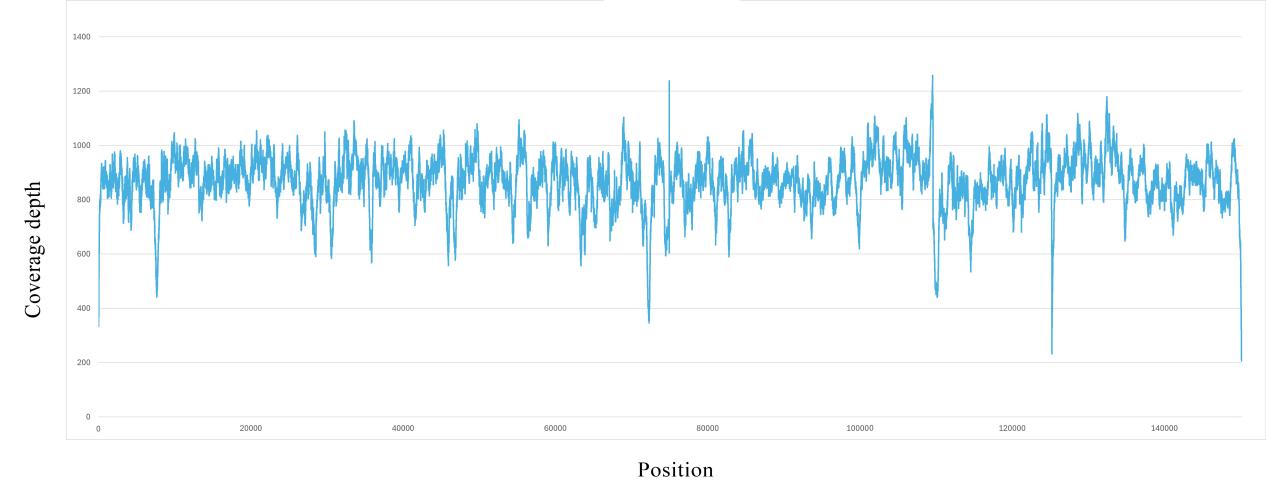

Supplement: Supplementary Figure 1 Read coverage plot.jpg [file TMDN_A_2432352_SM8061.jpg]
